# Efficient MIMO Configuration for Bi-Directional Vertical FSO Link with Multiple Beam Induced Pointing Error

**DOI:** 10.3390/s22239147

**Published:** 2022-11-25

**Authors:** Sung-Jin Kim, Sang-Kook Han

**Affiliations:** Department of Electrical and Electronic Engineering, Yonsei University, Seoul 03722, Republic of Korea

**Keywords:** atmospheric turbulence, diversity gain, free space optical communications, multi-input multi-output MIMO, pointing error, spatial diversity technique

## Abstract

We proposed the statistical misalignment model and the power-efficient configuration of transceivers for bi-directional multi-input and multi-output (MIMO) based vertical free space optical (FSO) links. Spatial diversity based MIMO FSO systems could be used to mitigate atmospheric fading issues. However, the increased number of channels can cause additional pointing error in pointing, acquisition and tracking (PAT) systems. The statistical misalignment model for detecting misalignment error is derived from the multiple transceivers. For the bi-directional characteristics of non-terrestrial back-haul networks, transmission performance is down-leveled to the worse in the asymmetric MIMO configuration of transceivers. The symmetric structure can mitigate the effect of increased pointing error to improve transmission performance. The proposed technique can be applied to the design of power-efficient FSO systems for non-terrestrial wireless back-haul networks.

## 1. Introduction

To provide network services everywhere, non-terrestrial networks have emerged as next-generation wireless back-haul systems. FSO communication has been actively studied for its application to non-terrestrial networks due to its great advantages of wide bandwidth, low power consumption, high security, etc. [1,2,3]. Unmanned aerial vehicle (UAV) based FSO systems suffer from atmospheric and misalignment induced fading. To overcome this atmospheric fading and connect areas hundreds of kilometers apart, high-altitude platform (HAP) UAVs are employed to establish FSO based back-haul networks. Although a horizontal FSO link can overcome the impact of atmospheric turbulence, a vertical FSO link still suffers from the atmospheric fading issue. These fading issues make the received power insufficient to establish seamless communication systems [4,5]. Therefore, improving the power margin is significant in order to achieve seamless and high data rate communication for FSO based non-terrestrial networks.

A well-known technique to mitigate channel fading issues is to utilize diversity channels. Time diversity and wavelength diversity are challenging to implement as they require long symbol duration and wide wavelength spacing [6,7]. However, a simple distant arrangement of a few meters can accomplish the required spatial diversity; and having a larger distance than the coherence length can easily achieve independent channels [8].

Many research groups have worked on spatial diversity based FSO systems. The effect of atmospheric turbulence on the MIMO based FSO communications was modeled by the Málaga distribution [9]. The outage probability of the multi-input single-output (MISO) and the MIMO FSO systems was analyzed with the atmospheric fading and the pointing error in the log-normally distributed channel [10,11]. The closed-form expression and performance analysis for the MIMO system were researched over the Gamma–Gamma distributed channels with a generalized pointing error in [12,13]. Moreover, the performance of spatial diversity based FSO systems was analyzed with the effect of non-zero boresight pointing errors [14,15]. The channel modeling with UAVs’ movements in the presence of atmospheric turbulence and pointing error was presented [16,17]. Although the impacts of various atmospheric channel models and pointing errors were analyzed for the MIMO FSO transmissions, these researches assumed that the pointing jitter is the only source of the pointing error. Thus, the pointing errors considered in these researches were consistent, regardless of the increasing number of channels.

However, the pointing error could be increased due to multiple beam transmissions in the misalignment detection process of PAT systems, so the pointing loss is increased to reduce the diversity gain. Therefore, even if the diversity performance is derived in the presence of atmospheric turbulence and pointing error, the achievable diversity gain could be worse than expected. The increased pointing error cannot be neglected to achieve the desired diversity gain using the spatial diversity technique. Our previous work [18] investigated a statistical misalignment model for the increased alignment error for multiple transmitters and a single receiver. We also analyzed how it is affected by atmospheric turbulence and the arrangement of the transmitters. However, when multiple receivers are employed, the increased pointing error can be reduced by averaging the detected misalignment. Furthermore, for the bi-directional communication characteristic of FSO based wireless back-haul networks, the pointing error behavior and the transmission performance of the uplink can differ from the downlink due to the reversed number of transmitters and receivers in the MIMO system. Thus, the transceiver configuration becomes significant for implementing power-efficient FSO transmissions.

In this paper, we propose the power-efficient MIMO configurations for the bi-directional vertical FSO link with the increased pointing error as the distant transceiver arrangement. The diversity gain is improved by minimizing the increased pointing error as the proposed system configuration. The proposed scheme can be utilized to achieve power-efficient transmissions for FSO based wireless back-haul networks.

## 2. FSO Channel Model

*M* transceivers at the ground terminal and *N* transceivers at the UAV terminal are considered for the MIMO based vertical FSO link.

### 2.1. Atmospheric Turbulence Channel

The atmospheric turbulence for the vertical FSO link becomes weaker as the altitude of transmissions increases, so we assumed the log-normally distributed channels for the weak turbulence regime as

(1) $$I^{mn}=e^{Z_{mn}},$$

where $Z_{mn}$ is a Gaussian random variable with the mean $\mu_{Z_{mn}}$ and the variance $\sigma_{Z_{mn}}^{2}$. Assume that $\mu_{Z_{mn}}=\mu_{Z}$ and $\sigma_{Z_{mn}}^{2}=\sigma_{Z}^{2}$ for all m∈{1,⋯,M} and n∈{1, ⋯, N} for the independent and identically distributed channels. The Rytov variance $\sigma_{Z}^{2}=-\mu_{Z}/2$ for each transceiver to ensure $Ε\{I^{m}\}=1$ is

(2) $$\sigma_{Z}^{2}=2.25k^{\frac{7}{6}}{\left(L-h_{0}\right)}^{\frac{5}{6}}\times \int_{h_{0}}^{L}C_{n}^{2}\left(l\right){\left(1-\frac{l-h_{0}}{L-h_{0}}\right)}^{\frac{5}{6}}{\left(\frac{l-h_{0}}{L-h_{0}}\right)}^{\frac{5}{6}}dl,$$

where k=2π/λ. λ, *L*, and $h_{0}$ are the wavelength, the altitude of the UAV, and the ground [19]. The perpendicular environment is assumed to make the transmission distance equal to the altitude of the UAV for the vertical link. The refractive index structure at altitude l follows the Hufnagel–Valley model, given by

(3) $$C_{n}^{2}\left(l\right)=0.00594{\left(V_{rms}/27\right)}^{2}{\left(10^{-5}l\right)}^{10}e^{-l/1000}+2.7\times 10^{-16}e^{-l/1500}+C_{n}^{2}\left(0\right)e^{-l/100},$$

where $V_{rms}$ is the rms wind speed and $C_{n}^{2}\left(0\right)$ is the refractive-index structure at the ground [19].

### 2.2. Pointing Error Model

Compared to MISO based FSO systems, the MIMO based FSO systems are assumed to have positioning sensors on each receiver side. The detected misalignments from each receiver are gathered and averaged to minimize the increased alignment error due to multiple transmissions. For *N* receivers to detect the misalignment with *M* transmitted beams, the transceivers are arranged to form a regular polygon with an equal distance between transceivers *d* to achieve the independent channel. We assumed that the transceivers are fixed in the same device with equivalent instantaneous pointing jitter, and the transmitted beams are directed to the center of the receiver arrangement. The detected misalignment for the *n*-th receiver is equivalent to the detected misalignment at the center of the receiver distribution with the translation by displacement of the receiver. The centroid of received multiple beams can be rewritten as the combination of the centroid $\Delta x_{mn}$ and the received power $I^{mn}$ of the *m*-th transmitted beam at the *n*-th receiver as described in [18]. Then, the aggregated horizontal centroid for the *n*-th receiver in MIMO transmissions can be expressed as

(4) $$\Delta x_{ac,n}=x_{n}+\frac{\sum_{m=1}^{M}\Delta x_{mn}\times I^{mn}}{\sum_{m=1}^{M}I^{mn}},$$

where $\Delta x_{ac,n}$ and $x_{n}$ are the aggregated centroid and displacement of the *n*-th receiver. The averaged centroid for *N* receivers is

(5) $$\Delta x_{ac}=\frac{1}{N}\sum_{n=1}^{N}\Delta x_{ac,n}=\frac{1}{N}\sum_{n=1}^{N}x_{n}+\frac{1}{N}\sum_{n=1}^{N}\left(\frac{\sum_{m=1}^{M}\Delta x_{mn}\times I^{mn}}{\sum_{m=1}^{M}I^{mn}}\right).$$

The receivers are also distributed with equal distance *d* to achieve the independent channel as the transmitter distribution. Thus, the sum of the receiver positions becomes the sum of the sines and cosines, which equals zero.

Note that the atmospheric channels for all diversity channels are independent of the distance between the transmitters or receivers and larger than the coherence length. Therefore, the detected misalignments for the *N* receivers are also independent. The sum of the log-normal random variable of received powers could be approximated to a single log-normal random variable using Wilkinson’s method [20]. Then, the averaged centroid in Equation (4) can be approximated as

(6) $$\Delta x_{ac}=\frac{1}{N}\sum_{n=1}^{N}\Delta x_{ac,n}=\frac{1}{N}\sum_{n=1}^{N}S,$$

where *S* is the Gaussian distributed statistical misalignment model with a zero mean and the variance $\sigma_{c,n}^{2}$ of aggregated centroid $\Delta x_{ac,n}$ as described in our previous work [18]. Since the sum of the Gaussian random variables is also the Gaussian random variable and the detected centroids for each receiver are independent, the mean of the averaged centroid is zero, and the variance of the overall detected misalignment error can be expressed as

(7) $$\sigma_{c}^{2}=\sum_{n=1}^{N}\frac{\sigma_{{}_{c,n}}^{2}}{N^{2}}\approx \frac{\left(e^{\sigma_{Z}^{2}}-1\right)d^{2}}{8\left(e^{\sigma_{Z}^{2}}-1+M\right)N\sin^{2}\left(\pi /M\right)}.$$

Note that the distance between the transmitter and the center of the transmitter arrangement is d/{2sin(π/M)}. The detected misalignment error is independent of the pointing jitter $\sigma_{s}^{2}$. Thus, the variance of the combined pointing error with increased misalignment and pointing jitter becomes $\sigma_{c}^{2}+\sigma_{s}^{2}$, and the pointing error model follows the Rayleigh distribution.

## 3. Diversity Performance

**Figure 1:**
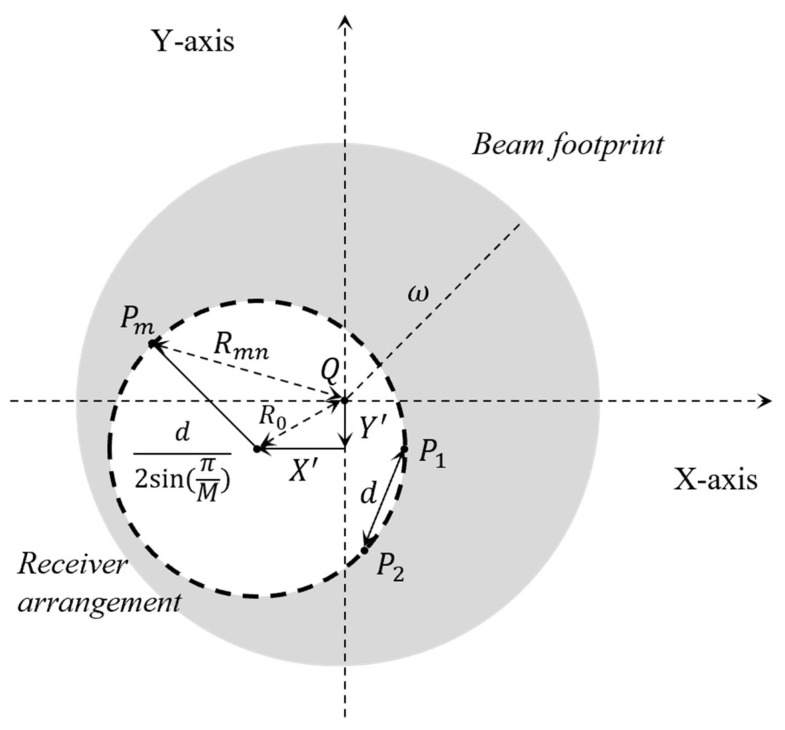
Beam footprint of the transmitter and receiver arrangement with pointing error.

Compared to MISO based systems, the MIMO systems have multiple *N* transceivers at the UAV. The misalignment is detected at the UAV with *M* transmitted beams from the ground and *N* positioning sensors. Then, the *N* transmitted beams with the pointing error propagate to *M* receivers at the ground terminal. Figure 1 shows the footprint of the transmitted beam and the arrangement of the *M* receivers at the ground. The pointed direction of the transmitted beam is assumed to aim at the center of the receiver distributions. The channel gain for M×N MIMO based FSO transmissions can be expressed as

(8) $$H=\sum_{m=1}^{M}\sum_{n=1}^{N}H_{mn}=\sum_{m=1}^{M}\sum_{n=1}^{N}A_{0}e^{Z_{mn}-2R_{mn}^{2}/\omega^{2}},$$

where ω and $R_{mn}$ are the beam width and the radial displacement of the *m*-th transmitted beam at the *n*-th receiver due to pointing error [11]. Because the pointing directions of the transmitted beams are aimed at the center of the receiver plane, the center of the beam footprints $Q_{n}$ are equal to *Q*, similar to the coordinates shown in Figure 1:

(9) $$Q_{n}=Q=\left[\begin{array}{c} X^{\prime }\\ Y^{\prime } \end{array}\right]\;\mathrm{for}\;\mathrm{all}\;n\in \left\{1,\;\cdots ,\;N\right\}.$$

$X^{\prime }$ and $Y^{\prime }$ are the displacements due to the pointing error. Thus, the combined channel gain for multiple transceivers in Equation (8) can be approximated with the random variables *G, T*, as referred to by [10] for MIMO transmissions:

(10) $$H=A_{0}e^{-2d^{2}/\left\{4\omega^{2}\sin^{2}\left(\pi /M\right)\right\}}e^{G-T},$$

where *G* is the Gaussian random variable with the mean $\mu_{G}$ and the variance $\sigma_{G}^{2}$ to represent the sum of the multiple atmospheric channels with the coordinates of transceivers as described in Appendix A. The beam parameters $A_{0}\approx 2a_{r}^{2}/\omega^{2}$. ω and $a_{r}$ are the beam width and the aperture size of the receiver. *T* is the random variable for the term of the pointing error, $T=2R_{0}^{2}/\omega^{2}$.

For the diversity performance analysis of MIMO based FSO systems, we assumed an intensity modulation and direct detection (IM/DD) system with non-return-to-zero on-off keying (NRZ-OOK) modulation. The outage of the transmitted signal is defined as the received power $P_{r}$ becoming less than the receiver sensitivity $P_{rs}$. Then, the outage probability from Equation (10) can be derived from the joint probability density function of *G* and *T* as referred to by [18]:

(11) $$\begin{array}{ll} p_{out}\left(P_{r}\right) & =\mathrm{Prob}\left(P_{r}<P_{rs}\right)=\mathrm{Prob}\left(H\cdot P_{t}<P_{rs}\right)\\ & =e^{\gamma^{4}\sigma_{G}^{2}/2-\gamma^{2}\mu_{G}}\times {\left(\frac{e^{2d^{2}/\left\{4\omega^{2}\sin^{2}\left(\pi /M\right)\right\}}}{A_{0}}\frac{P_{rs}}{P_{t}}\right)}^{\gamma^{2}} \end{array}$$

where $P_{t}$ is the transmitted power and γ is the beam parameter with the statistical misalignment model in Equation (7) given by

(12) $$\gamma =\frac{\omega }{2\sqrt{\sigma_{c}^{2}+\sigma_{s}^{2}}}.$$

The required power to achieve the target outage probability $p_{out}^{tg}$ for the system requirements can be derived from Equation (11) as

(13) $$P_{t}=\frac{P_{rs}}{A_{0}{\left(p_{out}^{tg}\right)}^{-\omega^{2}/\left\{4\left(\sigma_{s}^{2}+\sigma_{c}^{2}\right)\right\}}}e^{2d^{2}/\left\{4\omega^{2}\sin^{2}\left(\pi /M\right)\right\}+\omega^{2}\sigma_{G}^{2}/\left\{8\left(\sigma_{s}^{2}+\sigma_{c}^{2}\right)\right\}-\mu_{G}}.$$

## 4. Performance Analysis

The vertical FSO system as given in Table 1 is assumed to analyze the transmission performance with the simulation results. The vertical link distance is set to 20 km for a HAP-UAV operating in minimum wind speed. The receiver sensitivity for NRZ-OOK modulation is assumed to be −20 dBm to achieve a high data rate greater than tens of Gbps. The refractive structure at the ground is assumed to be 1 × 10^−12^ m^−2/3^ to analyze the effect of stronger atmospheric turbulence for the vertical FSO link. The distance between transmitters is set to 2 m to achieve spatially independent channels. The standard deviation of the pointing jitter is assumed to be 20 μrad from [21]. The detected misalignment error is analyzed for the transceiver configurations of the ground and the UAV terminals. The analysis of the transmission performance as the number of transceivers is presented for bi-directional communication FSO links.

### 4.1. Simulation Results for the Misalignment Model

**Figure 2:**
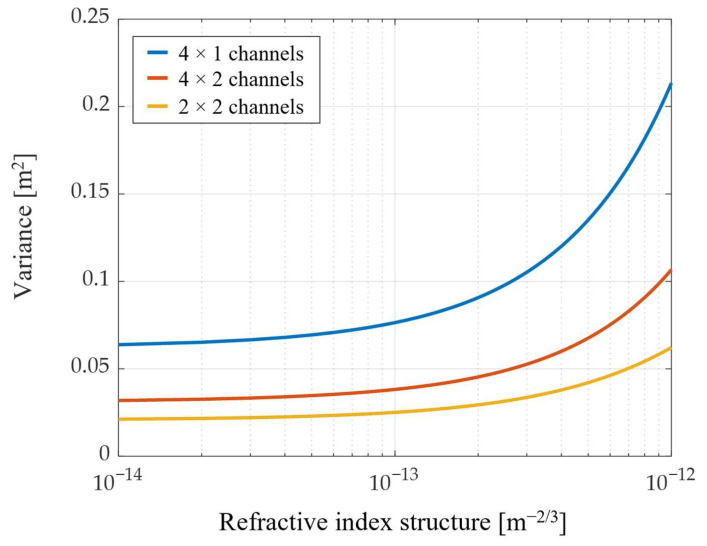
Variance of misalignment for different numbers of channels in MIMO transmissions.

Figure 2 shows the simulation results using Equation (7) for the MIMO transmissions with the different numbers of transmitters and receivers. To analyze the effect of different channels, the distance between transmitters was fixed to 2 m. The amount of misalignment was shown as the atmospheric turbulence strength became stronger with the different number of channels. The alignment error increases as the number of transmitters increases. In contrast, when the number of receivers increases, the alignment error decreases by averaging the detected misalignment. Thus, fewer transmitters and more receivers can reduce the increased alignment error due to multiple beam transmissions.

However, in the bi-directional communication characteristics of vertical FSO based back-haul networks, fewer transmitters and more receivers for one direction mean more transmitters and fewer receivers for the opposite direction. Therefore, the ratio between the transmitters and receivers for ground and UAV terminals becomes significant.

### 4.2. Transmission Performance

**Figure 3:**
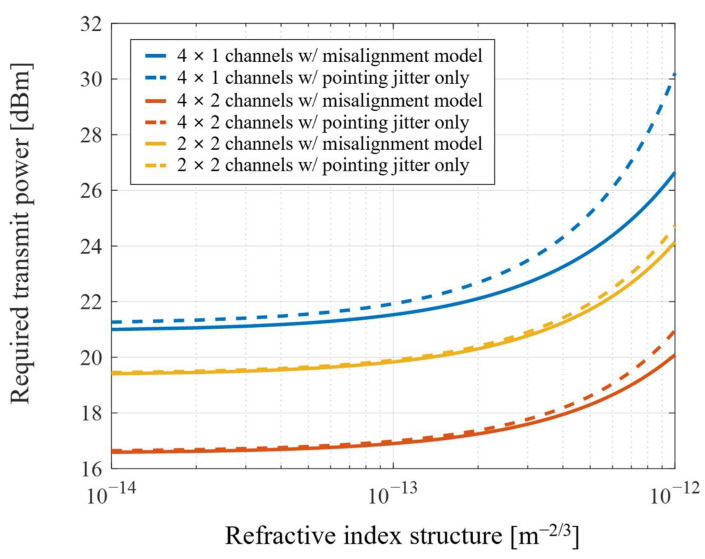
Required transmit power for the different number of channels.

The required transmission power to achieve the desired outage performance is shown in Figure 3. The transmission performances are compared for the systems designed by the proposed misalignment model and the pointing jitter as the only source of pointing error. The increase in pointing error for stronger turbulence requires more power to establish the communication links. Furthermore, more transmitted beams and fewer positioning sensors for misalignment detection make performance degradation severe as the pointing error increases.

To analyze the transmission performance for MIMO based FSO systems, the system configuration for bi-directional communication should be considered. For data reception at the ground terminal, the increased number of transceivers at the ground causes an increase in the incident beams in the positioning sensors at the UAV, which increases the detected misalignment. Furthermore, the number of transceivers at the ground makes the arrangement of transceivers larger. In contrast, the increased number of transceivers at the UAV causes the averaging effect in the detected misalignment to decrease the pointing error. However, the number of transceivers is still maintained, so the divergence loss does not change. These system configurations show better performance for the large number of transceivers at the UAV and the small number at the ground.

However, the system characteristics described above are reversed for data reception at the UAV terminal. The equivalent system configurations with the large number of transceivers at the UAV and the small number at the ground show poor performance due to increased divergence loss and pointing error. Therefore, the overall transmission performance of the bi-directional MIMO based FSO system is down-leveled to the worse performance.

**Figure 4:**
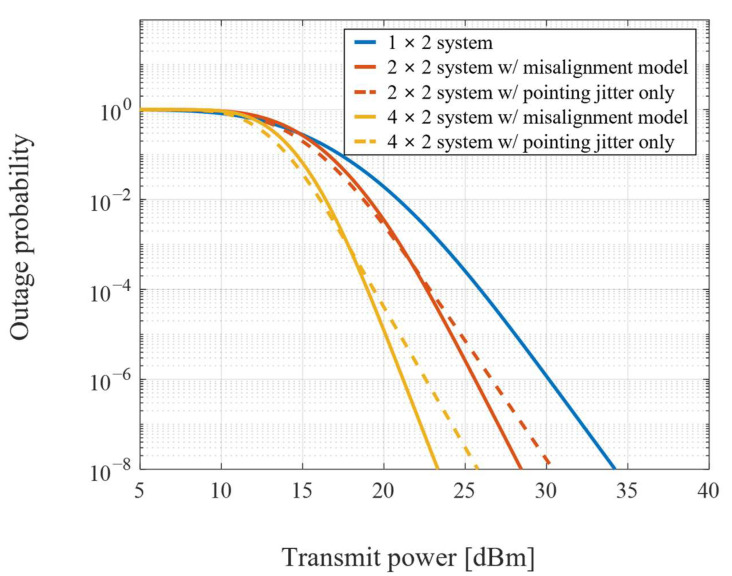
Outage performance of *M* × *N* systems for data reception at the ground terminal.

The outage performance for data reception at the ground terminal with two fixed transceivers at the UAV is shown in Figure 4. The 1 × 2 system has a single transmitted beam to detect misalignment, so the pointing error remains equivalent to the pointing jitter. The range of *M* receivers and the pointing error by the *M* transmitted beams to the UAV increase as the number of transceivers increases at the ground. Thus, the improvement of diversity gain becomes smaller as the pointing error increases by the total number of diversity channels.

**Figure 5:**
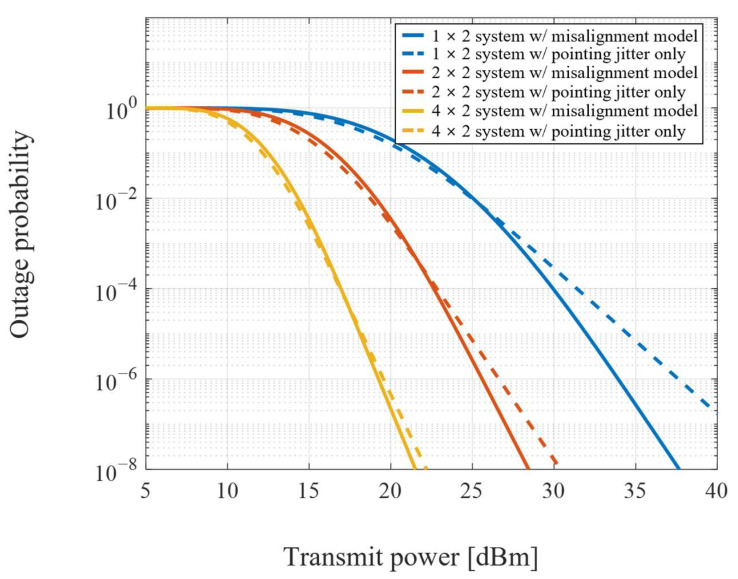
Outage performance of *M × N* systems for data reception at the UAV terminal.

Compared to the outage performance in Figure 4, the outage performance for data reception at the UAV is shown in Figure 5. The system characteristics are reversed due to the increasing number of *M* transceivers at the ground. The outage performance of 1 × 2 systems is worse than that of the ground due to the larger number of receivers at the UAV. However, the pointing error due to multiple beam transmissions decreases as the *M* positioning sensors at the ground increase. In contrast, the range of *N* receivers at the UAV is maintained. Thus, the improvement of diversity gain becomes larger with the number of channels. These results show an inversely proportional performance between the number of transceivers at the UAV and the ground. Therefore, the system configuration for bi-directional MIMO based FSO systems is significant.

**Figure 6:**
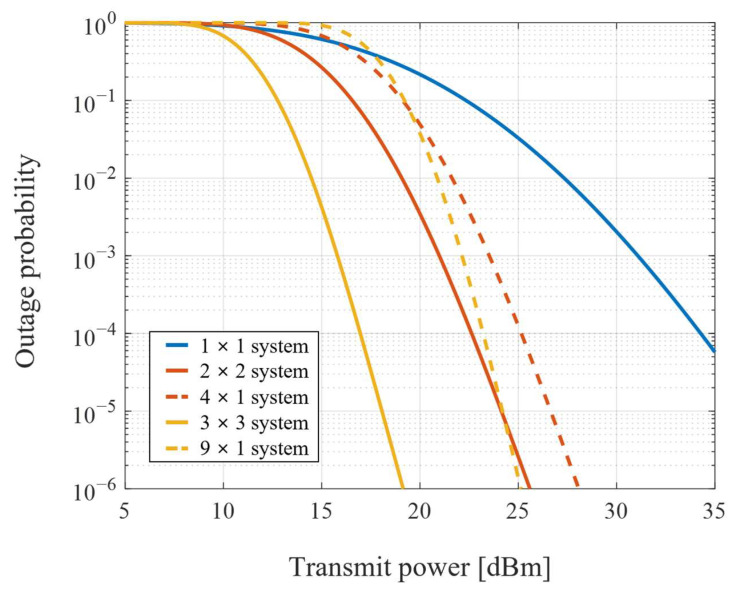
Overall outage performance of the total four and nine diversity channels.

The overall outage performance with the same total number of four and nine diversity channels is shown in Figure 6. It is difficult for the single-input single-output (SISO) based FSO system to achieve the desired performance due to limited transmission power in severe atmospheric turbulence. Thus, the spatial diversity technique is required to mitigate atmospheric fading by diversity gain. When the number of transceivers at the ground and UAV is different, the transmission performance of the system is down-leveled towards the worse. Therefore, the symmetric configuration of the transceivers at the UAV and ground terminals can minimize the effect of increased pointing error and achieve improved diversity gain.

## 5. Conclusions

In summary, we proposed the statistical misalignment model and the power-efficient system configuration for the MIMO based bi-directional vertical FSO systems. The detected misalignment increased with the number of transmitted beams and decreased with the number of positioning sensors. Considering the bi-directional nature of wireless back-haul networks, the effect of increased pointing error showed different results, and the transmission performance was restricted to the worse. Thus, the symmetric configuration of transceivers in the UAV and ground terminals can improve the achievable diversity gain by minimizing the increase in pointing error. The proposed technique can be used to design power-efficient FSO systems for non-terrestrial wireless back-haul networks for next-generation industries.

## Figures and Tables

**Table 1:** Vertical FSO link parameters.

Parameter	Value
Wavelength	1550 nm
Transmitter power	30 dBm
Receiver sensitivity	−20 dBm
Tx optics loss	−3 dB
Rx optics loss	−3 dB
Receiver aperture size	0.1 m
Link distance	20 km
Distance between transmitters	2 m
RMS wind speed	21 m/s
Refractive index structure on the ground	1 × 10^−12^ m^−2/3^
Standard deviation of pointing jitter	20 μrad
Target outage probability	1 × 10^−5^

## Data Availability

Not applicable.

## Appendix A

The sum of log-normally distributed channels with the displacement for each receiver is expressed as

(A1) $$e^{G}=\sum_{m=1}^{M}\sum_{n=1}^{N}e^{Z_{mn}-U_{mn}},$$

where

(A2) $$U_{mn}=\frac{4}{\omega^{2}}{\left[\begin{array}{c} X^{\prime }\\ Y^{\prime } \end{array}\right]}^{T}P_{m},$$

and $P_{m}$ is the position of the *m*-th receiver. The mean and variance of the Gaussian random variable *G* are referred to in [11] as

(A3) $$\mu_{G}=\log\left(\alpha \right)-\frac{\sigma_{G}^{2}}{2}\;\mathrm{and}\;\sigma_{G}^{2}=\log\left(1+\frac{\alpha^{2}}{\beta^{2}}\right),$$

where

(A4) $$\alpha =\sum_{k=1}^{K}e^{\mu_{k}+\frac{v_{kk}}{2}},$$

and

(A5) $$\beta^{2}=\sum_{k=1}^{K}\sum_{k^{\prime }}^{K}e^{\mu_{k}+\mu_{k^{\prime }}+\frac{v_{kk}+v_{k^{\prime }k^{\prime }}}{2}}\left(e^{v_{kk^{\prime }}}-1\right).$$

*K* is equal to M×N and k=ℳ(m, n) for all m∈{1, ⋯, M}, and n∈{1, ⋯, N}. ℳ(m, n) is a one-to-one mapping function from the pair (m, n) to an integer *k*.

After some mathematical calculation processes for Equations (A4) and (A5), with the system configuration included in Equation (9), as referred to in [10], the formulas α, and $\beta^{2}$ can be reorganized as

(A6) $$\alpha =MN\cdot e^{\frac{8}{\omega^{4}}\frac{\left(\sigma_{s}^{2}+\sigma_{c}^{2}\right)d^{2}}{4\sin^{2}\left(\pi /M\right)}},$$

and

(A7) $$\begin{array}{r} \beta^{2}=e^{{}^{\frac{8}{\omega^{4}}\left(\sigma_{s}^{2}+\sigma_{c}^{2}\right)d^{2}/\left\{4\sin^{2}\left(\pi /M\right)\right\}}}[MN\left\{e^{\sigma_{Z}^{2}}\left(\frac{16}{\omega^{4}}\frac{\left(\sigma_{s}^{2}+\sigma_{c}^{2}\right)d^{2}}{4\sin^{2}\left(\pi /M\right)}\right)+1\right\}\\ +N\frac{16}{\omega^{4}}\frac{\left(\sigma_{s}^{2}+\sigma_{c}^{2}\right)d^{2}}{4\sin^{2}\left(\pi /M\right)}\underset{-M}{\underset{︸}{\sum_{i=1}^{M-1}2\left(M-1\right)\cos\left(\frac{2\pi }{M}i\right)}}] \end{array}$$

Substituting Equations (A6) and (A7) into Equation (A3), the mean and variance can be approximated as

(A8) $$\sigma_{G}^{2}=\frac{1}{MN}\left(e^{\sigma_{Z}^{2}}-1\right)\;\mathrm{and}\;\mu_{G}=\log\left(MN\right)-\frac{\sigma_{G,MIMO}^{2}}{2}.$$
